# A Mumps Outbreak in Vojvodina, Serbia, in 2012 Underlines the Need for Additional Vaccination Opportunities for Young Adults

**DOI:** 10.1371/journal.pone.0139815

**Published:** 2015-10-23

**Authors:** Jasminka Nedeljković, Vesna Kovačević-Jovanović, Vesna Milošević, Zorica Šeguljev, Vladimir Petrovic, Claude P. Muller, Judith M. Hübschen

**Affiliations:** 1 Institute of Virology, Vaccine and Sera "Torlak", Belgrade, Serbia; 2 Institute of Public Health of Vojvodina, Centre for Disease Control and Prevention, Novi Sad, Serbia; 3 Department of Infection and Immunity, WHO-EURO Regional Reference Laboratory for Measles and Rubella, Luxembourg Institute of Health (former Centre de Recherche Public de la Santé) / Laboratoire National de Santé, Esch-Sur-Alzette, Grand-Duchy of Luxembourg; Alberta Provincial Laboratory for Public Health/ University of Alberta, CANADA

## Abstract

In 2012, mumps was introduced from Bosnia and Herzegovina to Vojvodina, causing an outbreak with 335 reported cases. The present manuscript analyses the epidemiological and laboratory characteristics of this outbreak, identifies its main causes and suggests potential future preventive measures. Sera of 133 patients were tested for mumps-specific antibodies by ELISA and 15 nose/throat swabs were investigated for mumps virus RNA by RT-PCR. IgG antibodies were found in 127 patients (95.5%). Mumps infection was laboratory-confirmed in 53 patients, including 44 IgM and 9 PCR positive cases. All other 282 cases were classified as epidemiologically-confirmed. More than half of the patients (n = 181, 54%) were 20–29 years old, followed by the 15–19 age bracket (n = 95, 28.4%). Twice as many males as females were affected (67% versus 33%). Disease complications were reported in 13 cases (3.9%), including 9 patients with orchitis and 4 with pancreatitis. According to medical records or anamnestic data, 190 patients (56.7%) were immunized with two doses and 35 (10.4%) with one dose of mumps-containing vaccine. The Serbian sequences corresponded to a minor genotype G variant detected during the 2011/2012 mumps outbreak in Bosnia and Herzegovina. Vaccine failures, the initial one-dose immunization policy and a vaccine shortage between 1999 and 2002 contributed to the outbreak. Additional vaccination opportunities should be offered to young adults during transition periods in their life trajectories.

## Introduction

Mumps is a contagious vaccine-preventable disease, caused by mumps virus (MuV), a member of the family *Paramyxoviridae*. The disease is generally mild, but in some cases can be associated with complications such as orchitis, encephalitis and deafness [1].

In Serbia, mumps has been a notifiable disease since 1978. Immunisation against mumps using measles-mumps (MM) vaccine was introduced in the childhood immunization schedule in 1986 (L-Zagreb strain; vaccine produced by the Institute of Immunology Zagreb). Since 1993 measles/mumps/rubella (MMR) vaccine, containing Urabe AM9 vaccine strain (mainly TRIMOVAX MÉRIEUX vaccine from Sanofi Pasteur), is used. An exception were the years 2001 and 2002 when the Jeryl Lynn strain in the Glaxo SmithKline MMR vaccine was applied. A two-dose schedule, with the first dose given at 12 months and the second at 12 years and no later than 14 years of age, was introduced in 1996. Since 2006, the second dose is administered at the age of 7 years [2].

In the Autonomous Province (AP) Vojvodina a large mumps outbreak occurred in 1988 with an incidence of 847 cases per 100,000 inhabitants [3]. This outbreak resulted in an increase of natural herd immunity in the population and a drastic decline in mumps incidence during the first few years of the immunization period. Between 1997 and 2006, the vaccination coverage for the first dose ranged from 82.1% to 98.1% with an average of 95.0%; the coverage for the second dose of mumps-containing vaccine ranged from 53.2% to 98.8% with an average of 87.1% [4]. The lowest second-dose coverage rates were recorded in 2002 (53.2%) and 2000 (62.0%), since between 1999 and 2002 there was a vaccine shortage all over Serbia due to importation problems. From 2003 until the outbreak in 2012, MMR coverage was continuously above 95% for both doses [5].

In 2012 a mumps outbreak involving 335 cases until the end of June occurred in AP Vojvodina. The present manuscript analyses the epidemiological and laboratory characteristics of this outbreak, identifies its main causes and suggests potential future preventive measures.

## Materials and Methods

### Ethics Statement

The physicians at the health centers were responsible for the clinical diagnosis of the parotitis cases. The investigation of this outbreak was done in the frame of non-research national public health surveillance for mumps and did not comprise any previously planned activities that could have been reviewed by an ethics committee or institutional review board. Sample collection was done for laboratory diagnosis as part of standard patient care and did therefore not require written informed consent. Clinical specimens were collected only if the patient provided oral consent.

The epidemiological staff of the Public Health Service of Vojvodina recorded patient data including date of birth, gender, place of residence, clinical symptoms, date of symptom onset, immunization status and disease complications. Data were reported on a weekly basis from the health centers to the Public Health Service of Vojvodina where descriptive epidemiological methods were used to process, evaluate and analyze all available patient data. Access to patient data was restricted to people directly involved in diagnosis and reporting to the treating physician.

### Case Definitions

According to the WHO criteria, a clinical case in the 2012 mumps outbreak was defined as a person with acute onset of unilateral or bilateral tender, self-limited swelling of the parotid or other salivary gland, lasting two or more days without other apparent cause [6]. A laboratory-confirmed case was defined as a clinical case of parotitis confirmed serologically and/or by molecular techniques. An epidemiologically confirmed case meets the clinical case definition and has an epidemiological link to a laboratory-confirmed case [6]. An imported case of mumps was defined as a clinical case epidemiologically linked to a mumps case in Bosnia and Herzegovina within the maximum length of the incubation period and an import-related case was defined as a person meeting the clinical criteria who has a direct epidemiological link to an imported case of mumps.

### Patient Samples

**Fig 1:**
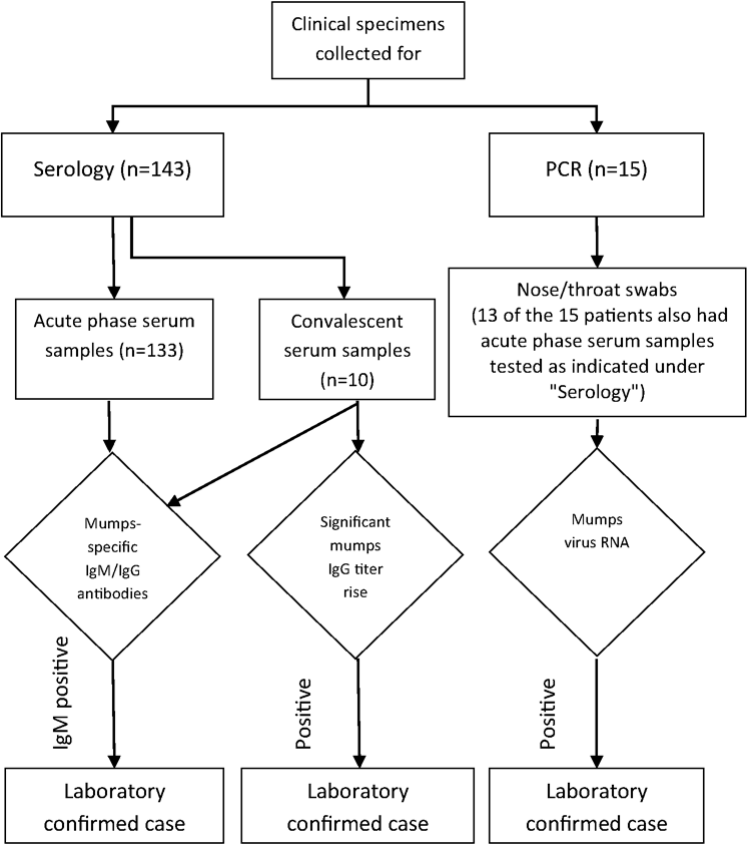
Testing algorithm of clinical samples.

Serum samples from 133 patients presenting with parotitis were collected 1–14 days after the onset of disease for serological diagnosis. Convalescent sera were obtained from 10 patients 7–14 days following the first serum sample. For molecular analysis nose/throat swabs were collected from 15 patients 1–5 days after disease onset and from 13 of these patients also serum samples were available for antibody detection (Fig 1).

### Serology

Sera of 133 patients were tested at the Institute of Public Health of Vojvodina. Determination of MuV specific IgM and IgG antibodies was done using EUROIMMUN AG IgM and IgG assays (Lübeck, Germany) according to the manufacturer’s instructions. The sera of the 13 patients of whom also nose/throat swabs were available, were investigated at Institute Torlak using Enzygnost anti-parotitis virus IgM and IgG kits (Siemens, Marburg, Germany) according to the manufacturer’s instructions.

### Molecular Diagnosis

Detection of MuV RNA was done by Real-time PCR at Institute Torlak. RNA was extracted from nose/throat samples using a QIAamp viral RNA kit (Qiagen, Hilden, Germany) according to the manufacturer’s instructions. For MuV detection a Real-time PCR was done using a Qiagen one-step RT-PCR kit (Hilden, Germany) and primers and probes provided by the Statens Institute, Denmark (MP1- f: 5'- CATAGGRGAYATGTGGGGACCAACCATT-3’; MP2 –r: 5'- GTCTTCGCCAACGATGGTGATGATTG-3’; MP probe: 5'-FAM-CCATGCAGGCGGTCACATTCCRACAACTGC-TAMRA-3’, Nielsen LP, Department of Microbiological Diagnostics and Virology, 2009, unpublished). PCR amplification of the SH gene region, sequencing, genotype determination and phylogenetic analysis were done at the WHO European Regional Reference Laboratory for Measles and Rubella in Luxembourg as described before [7]. The sequences are available under European Nucleotide Archive (ENA) accession numbers LN680922-9.

## Results

### Descriptive Epidemiology

The outbreak started with two mumps cases registered in Novi Sad, Serbia on 16^th^ January 2012 among students who had spent the Christmas and New Year holidays in Bosnia and Herzegovina, where a large outbreak of mumps was ongoing at that time [7,8]. The week after, another 15 cases who had spent their holidays in Bosnia and Herzegovina were notified. Subsequently, mumps spread from the imported cases to the population of Novi Sad. On January 25, the Public Health Service started to report mumps cases among persons living, working or studying in Novi Sad. These patients had not travelled outside the city during the maximum length of the incubation period [9], but were epidemiologically linked to imported mumps cases and thus considered as import-related cases. Eventually, the infection spread to other parts of the AP Vojvodina. The outbreak peaked between calendar weeks 11 and 14. By the end of June (week 26), 335 clinical mumps cases had been registered, among them 25 imported and 32 directly import-related cases. Although the main wave of the epidemic was over by June 30, 24 additional sporadic cases from other parts of Vojvodina were registered until the end of 2012. Disease complications were specifically reported only early during the outbreak in 13 patients (3.9%), including 9 patients with orchitis and 4 with pancreatitis.

**Fig 2:**
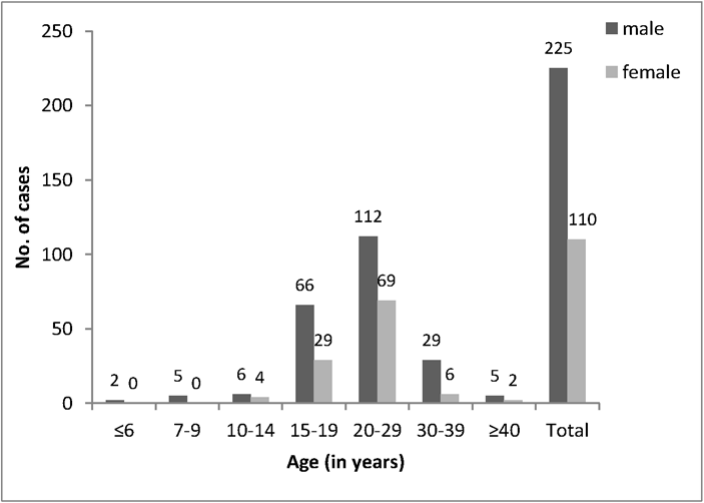
Number of mumps cases reported in Vojvodina between January and June 2012 according to age group and gender.

Of the 335 mumps cases, 225 (67%) were male and 110 (33%) were female (Fig 2). The age of the patients ranged between 4 and 58 years (mean 21.6 years). More than half of the patients (n = 181, 54%) were between 20 and 29 years old (Fig 2). The overall mumps incidence in AP Vojvodina was 17.3 per 100 000 inhabitants. The age-specific incidence rates ranged from 0.7 (age ≥40 years) to 86.5 (age 15–19 years) per 100 000.

Information about previous vaccinations was obtained from medical records in the health facilities (n = 196, 58.5%) or if unavailable from anamnestic data (n = 139, 41.5%). The medical records of students not originating in AP Vojvodina were kept in the health centers of their places of origin and the anamnestic data were in part collected by phone conversation of the students with their parents.

**Fig 3:**
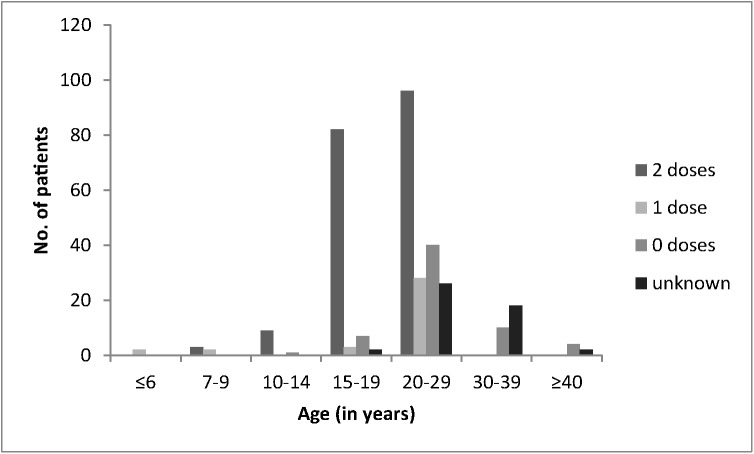
Mumps vaccination status of 335 cases reported in Vojvodina between January and June 2012 according to age group.

According to the medical records, a total of 169 patients had been vaccinated with two doses of MMR or MM vaccine and 27 with one dose (between 1986 and 1996 immunization was carried out with only one dose of mumps-containing vaccine). Among the 139 patients without records, 21 specified that they were immunized with two doses, 8 with one dose, 62 declared they had not received any mumps vaccine and 48 reported an unknown immunization status. Thus, 190 patients (56.7%) in total were considered immunized with two doses and 35 (10.4%) with one dose of mumps-containing vaccine; 62 (18.5%) had not received mumps vaccine and 48 (14.3%) had an unknown vaccination status. The majority of the patients with medical records and two doses of vaccine were between 15 and 29 years old (n = 157, 92.9%), while most of the patients with a single dose were between 20 and 29 years old (n = 20, 74.1%, Fig 3).

### Countermeasures

The public health authorities of AP Vojvodina recommended that the vaccination records of all children between one and 14 years of age should be checked and children who had not yet received the recommended MMR vaccine should be immunized. The public health authorities disseminated information about the mumps outbreak, infected persons were isolated as much as possible and people who had been in contact with mumps patients were placed under medical surveillance [9].

### Laboratory Findings

**Fig 4:**
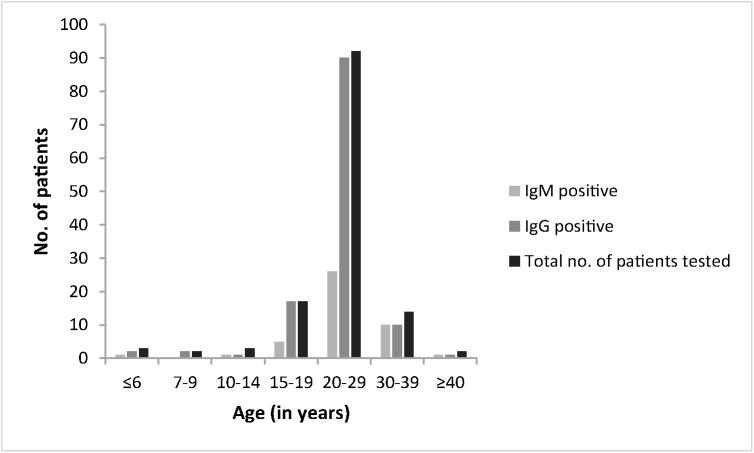
Serological status of 133 mumps patients reported in Vojvodina between January and June 2012 according to age group.

Mumps-specific IgM antibodies were detected in 44 out of 133 patients (33.1%), while IgG antibodies were found in 127 patients (95.5%). The highest proportion of IgM positives was detected among patients of at least 30 years of age (Fig 4).

**Fig 5:**
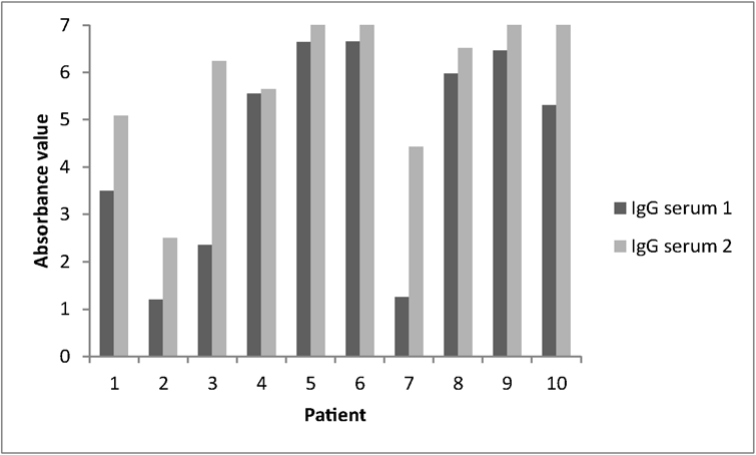
Mumps-specific IgG antibody absorbance values of 10 patients with convalescent sera from Vojvodina reported between January and June 2012.

In 2 of the 10 patients with convalescent serum, mumps-specific IgM antibodies were detected at the second collection time point (patient 7 absorbance value 5,45 and patient 10 absorbance value 1,12). All patients were already IgG positive at the initial testing and did not show a significant rise of antibody titers in the follow-up sample (Fig 5). No samples for PCR investigation were available from the 8 IgM negative patients.

Mumps-specific IgM antibodies were detected in 4 out of the six patients with orchitis tested (Table 1). Three of these IgM positive patients were unvaccinated and one had received a single dose of vaccine. The two investigated cases with pancreatitis were IgM negative and IgG positive (Table 1). All three patients vaccinated with two doses of mumps-containing vaccine were IgM negative, but IgG positive.

**Table 1:** Age, vaccinal status and serology results of 9 mumps patients with complications reported from Vojvodina between January and June 2012.

Patient	Complication	Age (in year)	Vaccinal status	IgM	IgG
11	orchitis	29	I dose	+	+
12	orchitis	20	non immunized	+	+
13	orchitis	18	**II** doses	-^a^	+ ^a^
14	orchitis	25	**II** doses	-	+
15	orchitis	32	non immunized	+ ^a^	+ ^a^
16	orchitis	28	non immunized	+	+
17	orchitis	37	non immunized	not done	not done
18	pancreatitis	19	II doses	-	+
19	pancreatitis	20	unknown	-	+

^a^ paired serum samples, results relate to second serum

Mumps virus RNA was detected in 9 of 15 patients (60%, Table 2). All PCR positive patients for whom serology results were available were IgM negative and IgG positive. Four RNA positive patients had been vaccinated with two doses, 2 had been vaccinated with one dose, another 2 had an unknown vaccination status and 1 patient had not received any mumps-containing vaccine.

**Table 2:** Laboratory results and characteristics of 15 mumps patients with specimens for molecular detection.

Patient	Age (in years)	Days between symptom onset and sample collection	ELISA results	Real-time PCR	Vaccination status
20	21	1	IgM-, IgG+	positive	two doses
21	20	2	IgM-, IgG+	positive	one dose
22	21	2	IgM-, IgG+	positive	unknown
23	21	2	IgM-, IgG+	positive	unknown
24	28	3	IgM-, IgG+	negative	two doses
25	22	4	IgM-, IgG+	negative	two doses
26	21	2	IgM-, IgG+	positive	two doses
27	4	3	IgM-, IgG+	negative	unknown
28	20	no data	IgM-, IgG+	negative	two doses
29	25	3	IgM-, IgG+	positive	unvaccinated
30	21	no data	IgM-, IgG ev^a^	negative	unknown
31	32	no data	not done	negative	unknown
32	7	2	IgM-, IgG+	positive	two doses
33	21	2	IgM-, IgG+	positive	two doses
34	22	3	not done	positive	one dose

^a^ equivocal

Overall, mumps infection was laboratory confirmed in 53 cases, including 44 IgM positive and 9 PCR positive cases. All other mumps cases were classified as epidemiologically confirmed (Table 3).

**Table 3:** Classification of all 335 mumps cases reported from Vojvodina between January and June 2012.

Method used for confirmation	Number of confirmed cases
Serology	44
Real-Time PCR	9*
Epidemiology	282
Total	335

*no IgM positives among them

**Fig 6:**
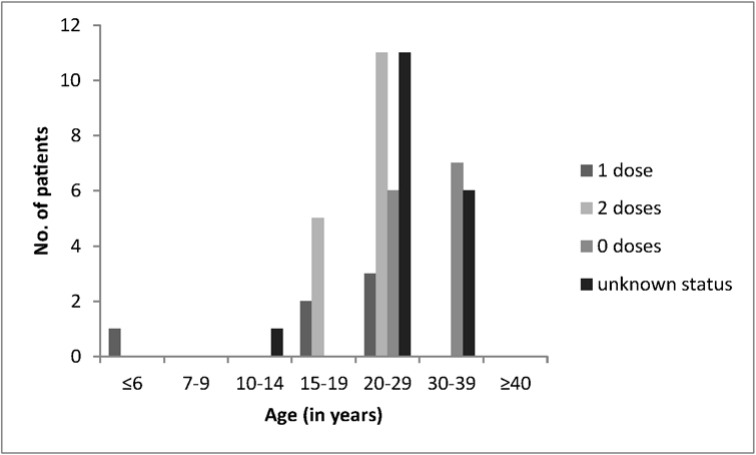
Immunization status of 53 laboratory confirmed mumps cases from Vojvodina reported between January and June 2012 according to age groups.

According to the medical records and anamnestic data, 16 of these 53 patients (30.2%) had been immunized with two doses and 6 (11.3%) with one dose of mumps-containing vaccine (Fig 6). Especially among the 20–39 year-old laboratory-confirmed cases were many with unknown vaccination status (n = 17) or not vaccinated (n = 13, Fig 6).

Amplification and sequencing of the mumps virus SH gene was successful for 8 of the 9 samples positive in the diagnostic PCR. All sequences were identical in the 316 nucleotide region used for genotyping (except for sequence MuVs/Novi Sad.SRB/3.12 showing a Y instead of a T at position 28) and corresponded to a minor genotype G variant detected during the mumps outbreak in Bosnia and Herzegovina (MuVs/Sarajevo.BIH/3.12/3, HF912221). No other identical sequences were identified using BLAST.

## Discussion

Despite a considerable reduction in mumps cases following the introduction of two-dose immunization programs, outbreaks continue to occur throughout Europe [7,8,10–12]. Unlike during the prevaccination period when mostly 5 to 9 year old children were affected, today mumps develops mainly in adolescents and adults, vaccinated or not during childhood [13,14]. Also in the present outbreak the most affected were the 15 to 29 year olds. The 20 to 29 year old people were supposed to get their first dose of vaccine between 1984 and 1993. However, vaccination against mumps started in Serbia only in 1986 and until 1996 included only a single dose of mumps-containing vaccine. Due to vaccine shortage between 1999 and 2002 throughout Serbia, coverage rates especially of the second dose dropped considerably and affected people who at that time were 12 years old and during the current outbreak between 22 and 25 years old. On the other hand, the majority of the adolescents and young adults with medical records (96.4% of the 15–19 and 79.4% of the 20–29 year olds) had received two doses of mumps vaccine (Fig 3). While this is in contrast to a recent mumps outbreak in Bosnia and Herzegovina where most of the reported cases were of similar age, but were not vaccinated or had an unknown vaccination history [7,8], mumps outbreaks in vaccinated populations have been described before [14–16]. Besides primary vaccine failures characterized by a lack of IgG antibody production, secondary vaccine failures with waning of antibodies and immunity have been reported [17,18]. Some authors observed a decline in protection with increasing age, but after 2 doses, vaccine effectiveness was reported to be above 85% even 6–7 years after the second vaccination [18]. It has been suggested that mumps manifests especially in cohorts which had received the latest (second) dose more than 10 years before [15]. This was the case for people older than 22 years at the time of the outbreak in Vojvodina. Compared to other European countries, Serbian children were with 12 years older when they received the second vaccine dose. Although this may be considered an advantage, it has been reported that mean antibody titers were somewhat lower before and after revaccination of 11- to 13-year-old children compared to the 4 to 6-year-olds [19].

In the present study, 41.5% of 53 laboratory confirmed mumps cases had been vaccinated, 16 patients with two doses and 6 patients with a single dose. Booster immune responses in patients with mumps secondary vaccine failure have been reported before [20] and we also observed high levels of mumps IgG antibodies in the six previously vaccinated RNA positive patients. While some of the IgM negative results may be explained by the partly early collection time point after symptom onset, our results showed, similar to reports from many other authors [21–23], that specific IgM antibodies are an unreliable marker of mumps infection in highly vaccinated populations. RNA detection proved helpful to confirm mumps in IgM negative patients with clinical parotitis. The overall low number of RNA positives in the present study could be due to inadequate sample collection and the storage of the swabs at -20°C before transportation to the laboratory.

The first mumps cases registered in AP Vojvodina were imported from Bosnia and Herzegovina where a large outbreak of mumps was ongoing at the time [7,8]. Interestingly, the sequences detected in Serbia were identical to a minor variant found only once among 57 strains reported from Bosnia and Herzegovina and no other identical sequences were found on GenBank. Further epidemiological investigation showed that the first patients came from the Republika Srpska in Bosnia and Herzegovina, from where no mumps strain information became available despite about 7700 registered cases during the 2011/2012 outbreak [7].

The complication rate at least early during the outbreak (13/119, 10.9%) [9] was slightly higher than previously reported [16,24,25], possibly because of the high numbers of affected postpubertal males. The more important disease complications in males [26] may also explain why overall twice as many male than female cases were reported.

In summary, the present outbreak may in part be due to vaccine failures, one dose immunization policy at the start of the mumps immunization programme, vaccine shortage between 1999 and 2002 and facilitated virus spread in high school and university settings. It is unclear whether additional factors such as vaccine cold chain breaches, differences in antibody titer and waning related to the vaccine used or genotypic mismatches between the vaccine strain and wildtype viruses circulating in the population played a role. Mumps outbreaks in vaccinated populations raise the issue of vaccine efficacy, and duration and robustness of protection. The reshuffling of birth cohorts with different levels of immunity warrants additional vaccination opportunities especially for young people at the transition of their life trajectories.
